# Comparison of Outcomes of Kidney Transplantation From Extremely Low Body Weight ≤5kg *Versus* Larger Body Weight Pediatric Donors

**DOI:** 10.3389/fimmu.2021.738749

**Published:** 2021-08-31

**Authors:** Jiawei Peng, Helong Dai, Hedong Zhang, Shaojie Yu, Xubiao Xie, Fenghua Peng, Gongbin Lan, Shanbiao Hu, Yu Wang, Xiaotian Tang, Yong Guo, Chen Gao, Chunhua Fang, Manhua Nie, Xiaoqiong Yuan, Mingda Zhong, Zhouqi Tang, Tengfang Li, Siyu Sun, Hengchang Yao, Jianfei Hou, Ruixue Huang, Longkai Peng

**Affiliations:** ^1^Department of Kidney Transplantation, The Second Xiangya Hospital of Central South University, Changsha, China; ^2^Clinical Research Center for Organ Transplantation in Hunan Province, Changsha, China; ^3^Clinical Immunology Center, Central South University, Changsha, China; ^4^Department of Occupational and Environmental Health, Xiangya School of Public Health, Central South University, Changsha, China

**Keywords:** center experience, kidney transplantation, extremely low body weight, pediatric donor, graft survival, utilization

## Abstract

**Background:**

Kidney transplantation from donors who weigh ≤5 kg is performed at only a few transplant centers owing to the high complication and low graft survival rates associated with this approach.

**Methods:**

We retrospectively compared the results of kidney transplantation at our center between January 2015 and December 2019 based on the following pediatric donor criteria: donor body weight ≤5 kg (n=32), 5 kg< donor weight ≤20 kg (n=143), and donor weight >20 kg (n=110). We also perform subgroup analysis of kidney transplantation outcomes from ≤5 kg donors, using conventional (dual separate and classic en-bloc KTx)/novel (en-bloc KTx with outflow tract) surgical methods and allocating to adult/pediatric recipients.

**Results:**

The death-censored graft survival rates from extremely low body weight ≤5kg at 1 month, and 1, 3, and 5 years were 90.6%, 80.9%, 77.5%, and 73.9%, respectively, which were significantly lower than that from larger body weight pediatric donors. However, the 3-, and 5-year post-transplantation eGFRs were not significantly different between the pediatric and adult recipient group. The thrombosis (18.8%) and urinary leakage (18.8%) rates were significantly higher in the donor weight ≤5 kg group. Compared with 5 kg< donor weight ≤20 kg group, donor weight ≤5kg group was at elevated risk of graft loss due to thrombosis (OR: 13.4) and acute rejection (OR: 6.7). No significant difference on the outcomes of extremely low body weight donor kidney transplantation was observed between adults and pediatric recipients. Urinary leakage rate is significantly lower in the novel operation (8.7%) than in the conventional operation group (44.4%).

**Conclusions:**

Although the outcomes of donor body weight ≤5kg kidney transplantation is inferior to that from donors with large body weight, it can be improved through technical improvement. Donors with body weight ≤5 kg can be considered as an useful source to expand the donor pool.

## Introduction

The number of patients diagnosed with end-stage kidney disease continues to increase worldwide, and dialysis or kidney transplantation is the therapeutic option available in such cases. Kidney transplantation, considered the treatment of choice in some patients with end-stage renal disease, is superior to dialysis with regard to patient survival and quality of life and is associated with better cognitive and mood regulation outcomes than those observed in patients who undergo dialysis (1–3). However, the demand for donor organs significantly exceeds the supply. Use of transplants from pediatric donors of extremely low body weight (≤5 kg) is viewed as a strategy to expand the donor pool.

Kidney transplantation using grafts obtained from pediatric donors of extremely low body weight is performed at only a few transplant centers, because pediatric donor kidney transplantation is associated with high rates of thrombotic and urinary tract complications, as well as acute rejection, delayed graft function (DGF), and hyperfiltration injury (4–9), particularly in cases of donors who weigh ≤5 kg (10–13). In this study, we performed an intergroup comparison of kidney transplantation outcomes based on the following pediatric donor criteria: donor body weight ≤5 kg, 5 kg< donor body weight ≤20 kg, and donor weight >20 kg. Additionally, we performed two subgroup analyses in the donor body weight ≤5 kg group, using conventional/novel surgical methods and allocating to adult/pediatric recipients.

## Materials and Methods

### Data Source

**Figure 1:**
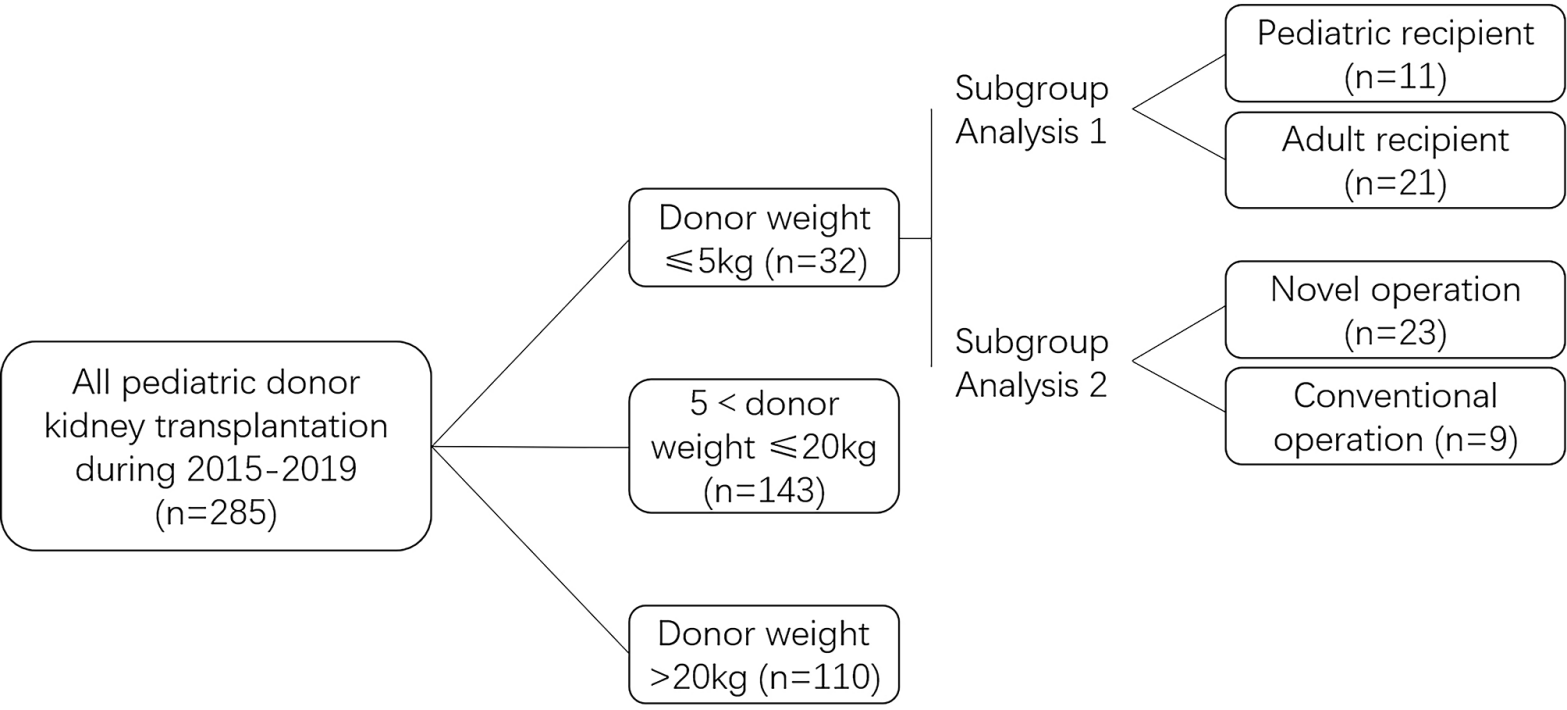
Study design.

We retrospectively investigated 32 patients who underwent kidney transplantation using grafts obtained from infants of extremely low body weight (≤5 kg) at our center between January 2015 and December 2019 and compared our results with those in patients who received kidneys from other pediatric donors during the same period (**Figure 1**). The control group was classified into the following groups: 5 kg <donor body weight ≤20 kg and donor body weight >20 kg. The donor body weight ≤5 kg group was subcategorized into pediatric and adult recipient groups for subgroup analysis. Based on different surgical techniques used, the donor body weight ≤5 kg group was subcategorized into novel operation and conventional operation groups (classical en-bloc kidney transplantation and dual separating kidney transplantation) for subgroup analysis. All study procedures were approved by the Ethics Committee of the Second Xiangya Hospital of Central South University.

### Donor and Recipient Selection

All donors in this study were aged <18 years. All donors in the experimental group (Group 1) weighed ≤5 kg. The control group included donors with the following body weight criteria: 5 kg <donor body weight ≤20 kg (Group 2) and donor body weight >20 kg (Group 3). All kidneys were obtained from deceased donors in accordance with the “Procedures and Standards for Organ Donation after the Death of Chinese Citizens”. The types of organ donation include DBD (donation after brain death) and DCD (donation after circulatory death). All donations were obtained after informed consent signed by guardians. All recipients were informed of the risks associated with pediatric donor kidney transplantation (14).

### Surgical Technique

Classical en-bloc kidney transplantation: We performed end-to-side anastomosis of the proximal end of the donor vena cava to the recipient’s external iliac artery and end-to-side anastomosis of the proximal end of the abdominal aorta to the recipient’s common iliac or external iliac artery. The distal end of the abdominal aorta and the inferior vena cava were ligated, and the bilateral ureters were successively anastomosed to the bladder apex using the Lich-Gregoir technique (15).

**Figure 2:**
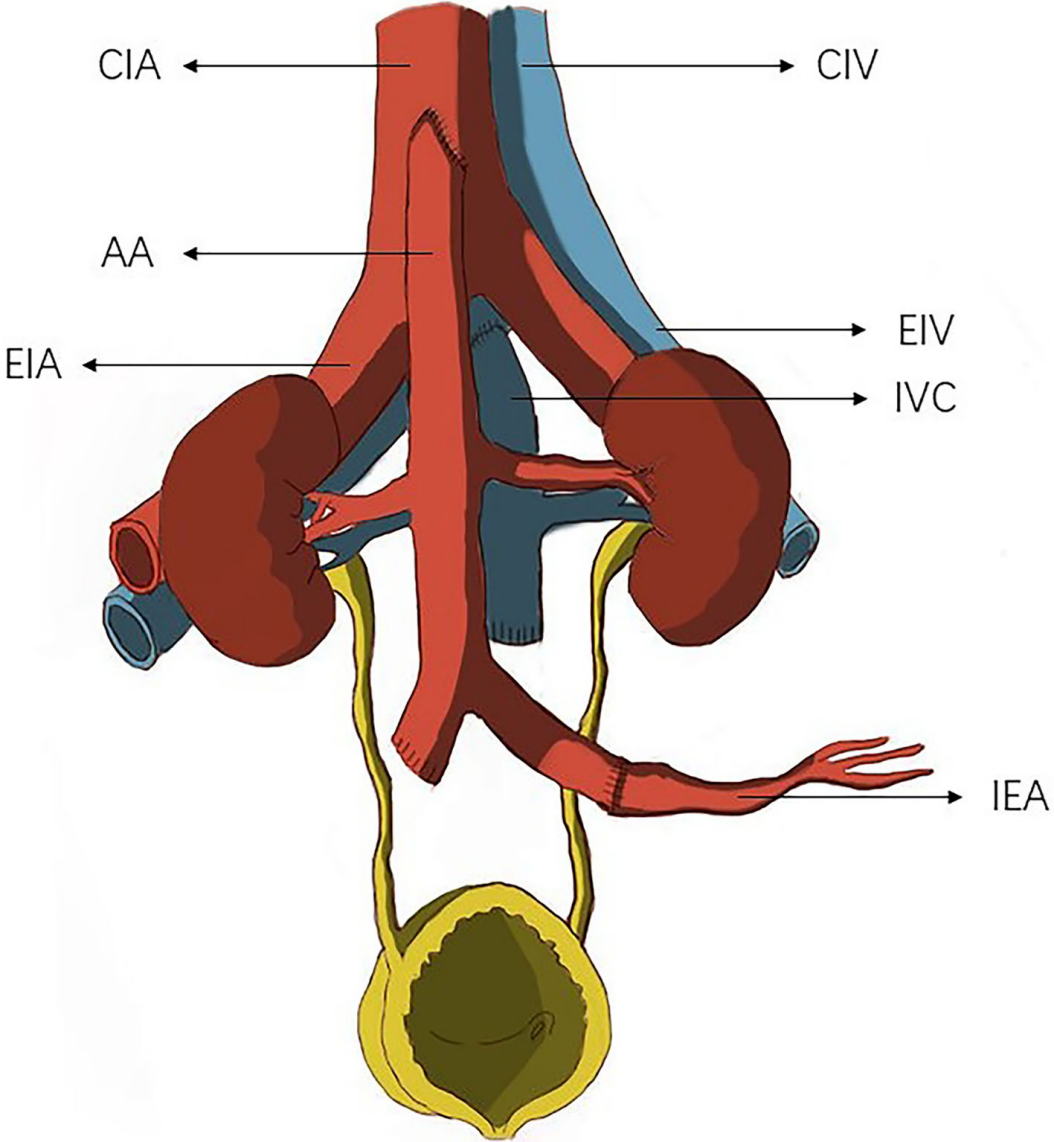
The donor common iliac artery or external iliac artery was anastomosed to the recipient inferior epigastric artery to establish an outflow tract. CIA, common iliac. artery; AA, abdominal aorta; EIA, external iliac artery; CIV, common iliac vein; EIV, external iliac vein; IVC, inferior vena cava; IEA, inferior epigastric artery.

Our novel technique improved the classical en-bloc kidney transplantation method using the distal abdominal aorta to establish an outflow tract (**Figure 2**): We performed end-to-side anastomosis of the proximal end of the donor renal inferior vena cava to the external iliac vein, and the distal end of the inferior vena cava was ligated. We performed end-to-side anastomosis of the proximal end of the aorta to the common iliac or external iliac artery and end-to-end anastomosis of the distal abdominal aorta of the donor to the inferior epigastric artery of the recipient to establish an outflow tract. The bilateral ureters were anastomosed to the recipient’s bladder (16).

Dual separating kidney transplantation: The left and right kidneys were separated and successively implanted into the ipsilateral iliac fossa. We performed end-to-side anastomosis of the renal veins of both grafts to the external iliac vein and end-to-end anastomosis of the renal artery to the internal iliac artery or end-to-side anastomosis of the renal artery to the external iliac artery. We performed end-to-side anastomosis of the renal artery of the distal graft to the external iliac artery, and the bilateral ureters were anastomosed to the bladder apex (15).

Single kidney transplantation: We performed end-to-side anastomosis of the graft renal vein to the external iliac vein, end-to-end anastomosis of the graft renal artery to the internal iliac artery or end-to-side anastomosis to the external iliac artery, based on the specific vascular conditions. The ureter was anastomosed to the bladder.

### Immunosuppression

All patients received mycophenolate mofetil (MMF) (1 g) and intravenous methylprednisolone (500 mg) pre-transplantation. The dosage of Immunosuppressant drugs was reduced in some pediatric KTx recipients based on physicians’ individual experience. Antithymocyte globulin or basiliximab was used for induction therapy and tacrolimus, MMF, and methylprednisolone were administered post-transplantation. The valley concentration of tacrolimus was maintained at 7–9 ng/mL during the first 3 months and 6–8 ng/mL during the first year post-transplantation. MMF was administered as an oral dose of 0.75 g twice a day, and the MMF area under the curve was maintained at 30–60 mg · hour/L. Methylprednisolone was administered at an initial dose of 64 mg/day, which was reduced by 8 mg/day and was eventually maintained at 8–16 mg/day.

### Post-Transplant Management

All patients received post-transplantation antibiotic prophylaxis against infection. Patients’ blood pressure was strictly controlled, with early systolic blood pressure maintained at 120–160 mmHg. The systolic blood pressure was maintained at levels <180 mmHg to avoid graft rupture. Anticoagulation was given to 7 out of 23 patients in novel operation group and 6 out of 9 in conventional operation group base on the surgeon’s intraoperative graft reperfusion assessment. Low-molecular-weight heparin (4100 IU) was injected subcutaneously twice a day during the first 3 days postoperatively, and this medication was switched to oral anticoagulation therapy (aspirin 100 mg/day or rivaroxaban 5 mg/day), 3 days later. Medication doses were reduced or the medication was discontinued in patients with a risk of bleeding. Double-J stents were removed a month after transplantation.

### Statistical Analysis

All statistical analysis were performed using the SPSS software, version 22. Normally distributed continuous variables are expressed as means ± standard deviation and non-normally distributed continuous variables as medians (range). Quantitative data were compared using the one-way analysis of variance, Kruskal-Wallis rank sum test, Mann-Whitney U test, and the t-test. Qualitative data were analyzed using the chi-square test, and the log-rank test was used for intergroup comparison of the overall differences in survival curves. A *p* value <0.05 was considered statistically significant.

## Results

**Table 1** shows the demographic data of donors and recipients. No significant intergroup differences were observed in the cold ischemia time (CIT), warm ischemia time (WIT), pre-transplantation dialysis time, re-transplantation rates, and human leukocyte antigen mismatches. The age and body weight of donors and recipients and donor/recipient weight ratio were significantly lower in Group 1 than in Groups 2 and 3. The percentage of male donors and recipients was 40.6% and 46.9%, respectively in Group 1, which were lower than the percentages in groups 2 and 3.

**Table 1:** Donor and recipient demographics.

	Donor weight ≤5kg (n=32)	5<donor weight ≤20kg (n=143)		Donor weight >20kg (n=110)	P
Donor					
Age, year	0.08 (0.01-0.42)	2 (0.25-8)		14 (6-17)	0.000
Weight, kg	3.51 ± 0.77	12.79 ± 4.33		42.77 ± 13.69	0.000
Male, % (n)	40.6% (13)	69.2% (99)		61.8% (68)	0.009
CIT, hr	5.25 (2.1-12)	7 (2-12)		7 (1-13)	0.754
WIT, min	0 (0-13)	0 (0-12)		0 (0-12)	0.900
Recipient					
Age, y	27.56 ± 14.68	32.42 ± 12.73		36.34 ± 12.38	0.002
Weight, kg	44.11 ± 12.95	50.87 ± 12.24		59.78 ± 12.4	0.000
Donor/Recipient weight	0.08 (0.05-0.21)	0.23 (0.10-0.98)		0.70 (0.29-0.68)	0.000
Male, % (n)	46.9% (15)	53.1% (76)		77.3% (85)	0.000
Dialysis time, mon	11.5 (0-63)	12 (0-240)		12 (0-360)	0.500
Re-transplantation, % (n)					0.469
First	96.9% (31)	99.3% (142)		99.1% (109)	
Second	3.1% (1)	0.7% (1)		0.9% (1)	
HLA mismatch, n	2 (0-4)	2 (0-4)		2 (0-4)	0.125

CIT, Cold ischemia time; total time from aortic perfusion to re,perfusion of kidneys. WIT, Warm ischemia time; asystole to commencement of aortic perfusion. eGFR, Estimated glomerular filtration rate; HLA, Human leukocyte antigen.

**Figure 3:**
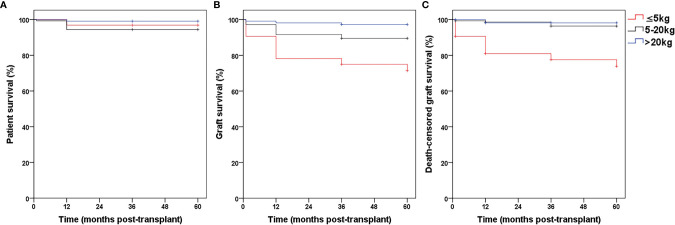
The patient survival (**A**, *P >* 0.05), graft survival (**B**, *P <* 0.05) and death-censored graft survival (**C**, *P <* 0.05) rates in Group 1, 2 and 3.

**Figure 4:**
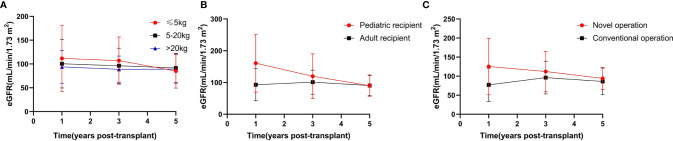
**(A)** The 1, 3, and 5 years eGFR in Group 1, 2 and 3, *P >* 0.05. **(B)** The 1, 3, and 5 years eGFR in Group pediatric recipients and adult recipients, *P* in 1 year < 0.05, *P* in 3 and 5 years > 0.05. **(C)** The 1, 3, and 5 years eGFR in Group novel operation and conventional, P > 0.05.

Patient survival rates at 1 month, as well as 1, 3, and 5 years were 100%, 96.9%, 96.9%, and 96.9%, respectively in Group 1, which did not significantly differ from those in Groups 2 and 3. The graft survival and death-censored graft survival rates at 1 month, as well as 1, 3, and 5 years were 90.6%, 78.1%, 75%, 71.4% and 90.6%, 80.9%, 77.5%, 73.9%, respectively in Group 1, which were significantly lower than those in Groups 2 and 3 (**Table 2** and **Figure 3**). No significant intergroup differences were observed in the 1-, 3-, and 5-year estimated glomerular filtration rate (eGFR) (**Figure 4A**). The thrombosis and urinary leakage rates were significantly higher in Group 1 than in Groups 2 and 3; however, we observed no significant intergroup differences in arterial stenosis, DGF, acute rejection, hydronephrosis, and ureteral stenosis rates.

**Table 2:** Comparison of kidney transplantation outcomes.

	Donor weight ≤5kg (n=32)	5<donor weight ≤20kg (n=143)	Donor weight >20kg (n=110)	*P*
Patient survival, %				0.132
1 mon	100%	99.30%	100%	
1 y	96.90%	94.44%	99.10%	
3 y	96.90%	94.44%	99.10%	
5 y	96.90%	94.44%	99.10%	
Graft survival, %				0.000
1 mon	90.60%	97.20%	99.10%	
1 y	78.10%	91.60%	98.20%	
3 y	75%	89.50%	97.30%	
5 y	71.40%	89.50%	97.30%	
Death-censored graft survival, %				0.000
1 mon	90.60%	99.30%	100%	
1 y	80.90%	98.60%	98.20%	
3 y	77.50%	96.40%	98.20%	
5 y	73.90%	96.40%	98.20%	
eGFR, mL/min/1.73 m^2^				
1y	111.81 ± 69.66	100.36 ± 50.68	93.85 ± 3.841	0.199
3 y	107.02 ± 9.70	96.252 ± .77	88.672 ± .85	0.070
5 y	85.335 ± .99	91.645 ± .57	89.325 ± .89	0.774
Complications, % (n)				
Vascular				
Stenosis	0	0.7% (1)	0.9% (1)	0.863
Thrombosis	18.8% (6)	2.1% (3)	0	0.000
Delayed graft function	34.4% (11)	23.1% (33)	16.4% (18)	0.081
Urine leak	18.8% (6)	4.9% (7)	1.8% (2)	0.001
Acute rejection	12.5% (4)	10.5% (15)	10.9% (12)	0.947
Hydronephrosis	3.1% (1)	0	0.9% (1)	0.151
Ureterostenosis	3.1% (1)	1.4% (2)	0.9% (1)	0.644

Graft loss was primarily attributable to thrombosis and acute rejection (**Table 3**). No significant differences in chronic allograft nephropathy and primary non function were observed between Groups 1, 2 and 3 as shown in **Table 3**. The odds ratio (OR) of thrombosis was 13.406-fold higher in Group 1 than in Group 2. Although no significant intergroup difference on acute rejection incidence is observed, acute rejection is inclined to cause graft loss in donor body weight ≤5kg group. The OR of acute rejection leading to graft loss was 6.703- and 10.313-fold higher in Group 1 than in Groups 2 and 3, respectively.

**Table 3:** Causes of graft losses.

	Donor weight ≤5kg (n=32)	5<donor weight ≤20kg (n=143)	Donor weight >20kg (n=110)	*P*
Thrombosis, % (n)	9.4% (3)	0.7% (1)	0	0.000
Acute rejection, % (n)	9.4% (3)	1.4% (2)	0.9% (1)	0.009
Chronic allograft nephropathy, % (n)	3.1% (1)	1.4% (2)	0.9% (1)	0.644
PNF, % (n)	0	0	0	
	**Donor weight ≤5kg (n=32)**	**5<donor weight ≤20kg (n=143)**	***P***	**OR**
Thrombosis, % (n)	9.4% (3)	0.7% (1)	0.02	13.406
Acute rejection, % (n)	9.4% (3)	1.4% (2)	0.043	6.703
Chronic allograft nephropathy, % (n)	3.1% (1)	1.4% (2)	0.456	
PNF, % (n)	0	0		
	**Donor weight ≤5kg (n=32)**	**Donor weight >20kg (n=110)**	***P***	**OR**
Thrombosis, % (n)	9.4% (3)	0	0.011	
Acute rejection, % (n)	9.4% (3)	0.9% (1)	0.036	10.313
Chronic allograft nephropathy, % (n)	3.1% (1)	0.9% (1)	0.401	
PNF, % (n)	0	0		

PNF, primary nonfunction; OR, Odds Ratio is used to reflect the difference in exposure factors between the experimental group and the control group, so as to establish the connection between disease and exposure factors.

**Figure 5:**
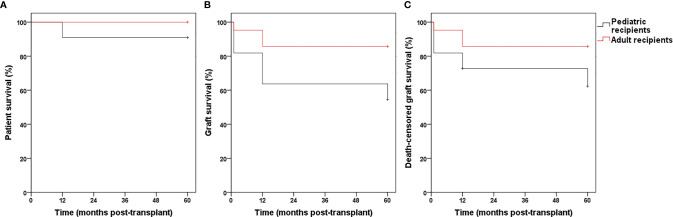
The patient survival (**A**, *P >* 0.05), graft survival (**B**, *P >* 0.05) and death-censored graft survival (**C**, *P >* 0.05) rates in Group pediatric recipients and adult recipients.

No significant differences were observed in age, sex, CIT and WIT of donors and sex of recipients and donor/recipient weight ratio between the pediatric recipient and adult recipient groups (**Table 4**). Donors’ weight was significantly lower in the pediatric recipient than in the adult recipient group due to donor/recipient body weight match principle. The patient survival, graft survival, and death-censored graft survival rates at 1 month, as well as 1, 3, and 5 years were statistically nonsignificant in the adult recipient and pediatric recipient group (**Figure 5**). The 1-year post-transplantation eGFR was significantly higher in the pediatric recipient than in the adult recipient group; however, the 3-, and 5-year post-transplantation eGFRs were not significantly different between these subgroups (**Figure 4B**). We observed no significant differences in thrombosis, DGF, urine leak, acute rejection, hydronephrosis, ureterostenosis.

**Table 4:** Subgroup comparison of kidney transplantation using grafts obtained from pediatric donors of extremely low body weight with different recipients.

	Pediatric recipient (n=11)	Adult recipient (n=21)	P
Donor			
Age, year	0.08 (0.01-0.33)	0.17 (0.01-0.42)	0.347
Weight, kg	3.00 ± 0.64	3.78 ± 0.71	0.005
Male, % (n)	36.4% (4)	42.9% (9)	1.000
CIT, hr	4.00 (3.00-12.00)	5.30 (2.10-11.0)	0.858
WIT, min	0.00 (0.00-7.00)	0.00 (0.00-11.00)	0.179
Recipient			
Age, y	11.36 ± 2.77	36.05 ± 10.51	0.000
Weight, kg	27.90 (14.30-54.50)	48.00 (41.20-64.30)	0.001
Donor/Recipient weight	0.11 ± 0.05	0.08 ± 0.02	0.057
Male, % (n)	72.7% (8)	33.3% (7)	0.062
Dialysis time, m	4.91 ± 4.09	20.33 ± 16.39	0.000
Patient survival, %			0.167
1mon	100%	100%	
1 y	90.90%	100%	
3 y	90.90%	100%	
5 y	90.90%	100%	
Graft survival, %			0.053
1mon	81.82%	95.24%	
1 y	63.64%	85.71%	
3 y	63.64%	85.71%	
5 y	54.55%	85.71%	
Death-censored graft survival, %			0.142
1 mon	81.82%	95.24%	
1 y	72.73%	85.71%	
3 y	72.73%	85.71%	
5 y	62%	85.71%	
eGFR, mL/min/1.73 m^2^			
1y	160.76 ± 91.03	92.77 ± 50.54	0.025
3 y	119.55 ± 70.91	101.18 ± 37.87	0.538
5 y	90.175 ± 34.16	90.405 ± 31.46	0.991
Complications, % (n)			
Thrombosis	36.4% (4)	9.5% (2)	0.148
Delayed graft function	36.4% (4)	33.3% (7)	1.000
Urine leak	9.1% (1)	23.8% (5)	0.637
Acute rejection	9.1% (1)	14.3% (3)	1.000
Hydronephrosis	0	4.8% (1)	1.000
Ureterostenosis	0	4.8% (1)	1.000

**Figure 6:**
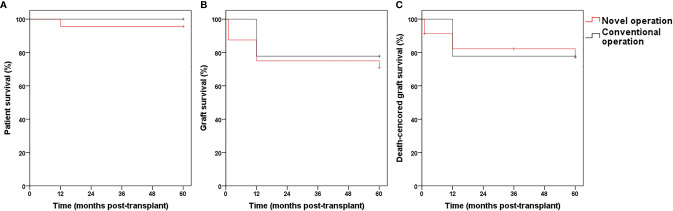
The patient survival (**A**, *P >*0.05), graft survival (**B**, *P >*0.05) and death-censored graft survival (**C**, *P >*0.05) rates in Group novel operation and conventional.

We observed no significant differences in donor weight and sex, as well as CIT and WIT and sex, and dialysis time of the recipients and donor/recipient weight ratio between the two subgroups (**Table 5**). Donor age and the age and weight of recipients were significantly lower in the novel operation than in the conventional operation group. We observed no significant intergroup differences in patient survival, graft survival, death-censored graft survival rates (**Figure 6**) and post-transplantation eGFR (**Figure 4C**). Urinary leakage rates were significantly lower in the novel operation than in the conventional operation group. The urine leakage rate in the novel operation group was 0.196-fold less than conventional operation group. The thrombosis, DGF, hydronephrosis, and ureteral stenosis rates were lower in the novel operation than in the conventional operation group; however, the difference was statistically nonsignificant. Therefore, it is reasonable to conclude that the novel operation scores over the conventional operation with regard to complication rates, although further accumulation of cases is essential to support these findings.

**Table 5:** Subgroup comparison of kidney transplantation using grafts obtained from pediatric donors of extremely low body weight with different surgical methods.

	Novel operation (n=23)	Conventional operation (n=9)	P	OR
Donor				
Age, year	0.06 (0.01-0.42)	0.17 (0.01-0.42)	0.047	
Weight, kg	3.41 ± 0.75	3.78 ± 0.8	0.226	
Male, % (n)	43.5% (10)	33.3% (3)	0.704	
CIT, hr	6.75 ± 3.1	5.44 ± 2.93	0.286	
WIT, min	0 (0-11)	0 (0-8)	0.579	
Recipient				
Age, y	23.43 ± 13.51	38.11 ± 12.63	0.009	
Weight, kg	40.68 ± 11.42	52.87 ± 13.06	0.014	
Donor/Recipient weight	0.08 (0.06-0.21)	0.07 (0.05-0.15)	0.271	
Male, % (n)	39.1% (9)	66.7% (6)	0.243	
Dialysis time, m	16.48 ± 17.47	11.33 ± 6.78	0.240	
Patient survival, %			0.532	
1mon	100%	100%		
1 y	95.65%	100%		
3 y	95.65%	100%		
5 y	95.65%	100%		
Graft survival, %			0.643	
1mon	86.96%	100%		
1 y	73.91%	77.78%		
3 y	73.91%	77.78%		
5 y	69.57%	77.78%		
Death-censored graft survival, %			0.926	
1 mon	91.30%	100%		
1 y	82.20%	77.80%		
3 y	82.20%	77.80%		
5 y	77%	77.80%		
eGFR, mL/min/1.73 m^2^				
1y	125.31 ± 74.07	77.09 ± 43.70	0.122	
3 y	112.12 ± 5.19	96.112 ± 42.89	0.495	
5 y	94.095 ± 28.80	86.065 ± 35.01	0.634	
Complications, % (n)				
Thrombosis	13% (3)	33.3% (3)	0.314	
Delayed graft function	26.1% (6)	55.6% (5)	0.213	
Urine leak	8.7% (2)	44.4% (4)	0.038	0.196
Acute rejection	13% (3)	11.1% (1)	0.689	
Hydronephrosis	0	11.1% (1)	0.281	
Ureterostenosis	0	11.1% (1)	0.281	

## Discussion

The widening gap between organ demand and supply is a worldwide concern. The infant mortality rate in China is ≥1.39% (13, 17). A survey has shown that young parents are more likely to consent to organ donation, following cardiac death in an infant (13). Although this is a good source to expand the donor pool, many low body weight ≤20kg donor kidneys are discarded (18, 19) owing to the general opinion that kidneys from low body weight donors are prone to thrombotic and urinary tract complications, acute rejection, DGF, and hyperfiltration injury (4–9). However, in recent times, research centers are increasingly using small pediatric donor kidneys, which have shown similar or better short-/long-term results compared with adult donor kidneys (20–22). Sharma et al. and Sureshkumar et al. compared deceased small pediatric donor kidneys with living donor kidneys and concluded that the long-term outcomes of pediatric en-bloc kidney transplantation using grafts from deceased donors are similar to or better than those of living donor kidneys, although early thrombosis remains a challenging complication associated with pediatric en-bloc kidney transplantation (23, 24). As previously mentioned, low body weight (≤20kg) pediatric donor kidneys can be utilized safely and effectively. However, when the pediatric donor weight comes down to ≤5kg, it gets considerably trickier.

Currently only a few research centers have reported the use of kidneys from donors who weigh ≤5 kg. Wijetunga et al. performed kidney transplantation in 15 patients who received kidneys from donors who weighed ≤5 kg and observed a 1-year graft survival rate of 86.7% with thrombosis in 3 patients (20%) (12). Sui et al. performed kidney transplantation in 10 patients who received kidneys from donors who weighed ≤5 kg and observed thrombosis in 3 patients (30%). Zhao et al. performed kidney transplantation in 4 patients who received kidneys from donors who weighed ≤5 kg, and observed thrombosis in 2 patients (25%) (13). We performed kidney transplantation in 32 patients who received grafts from donors who weighed ≤5 kg (the thrombosis rate was 18.8%) and compared with those using grafts from donors who weighed 5–20 kg and donors who weighed >20 kg. The eGFR and patient survival in Group 1 was not significantly different from that observed in groups that included patients of greater body weight. However, the graft survival and death-censored graft survival rate were significantly lower in Group 1 than in Groups 2 and 3, and the thrombosis and urinary leakage rates were significantly higher in Group 1 than in Groups 2 and 3, which may be attributable to the long learning curve of kidney transplantation surgery using organs from infants of extremely low body weight, the fact that we did not select the appropriate surgical methods during the early stages, and smaller blood vessels may have predisposed patients to thrombosis and graft loss.

Some research centers recommend that pediatric donor kidneys primarily be used in children and this practice is associated with favorable outcomes (5, 23, 25–28). However, others are of the view that pediatric donor kidneys should primarily be used in adult recipients (29–32). This recommendation is based on the fact that the total number of nephrons in the kidney (approximately 1 million) is determined in utero, and no new nephrons are formed after 36 weeks of gestation (33); therefore, the number of renal units in pediatric donor kidneys is the same as those in adult donor kidneys, with a difference only in their size. Based on an individual’s requirements, a pediatric donor kidney shows compensatory hypertrophy after transplantation in an adult, whereas the pediatric donor kidney tends to grow slowly in pediatric recipients (6, 28, 34–36). We observed that 1month, as well as 1, 3, and 5 years post-transplantation patient survival, graft survival, and death-censored graft survival rates were statistically nonsignificant between pediatric and adult recipients. Although the 1-year post-transplantation eGFR was significantly higher in the pediatric recipient than in the adult recipient group, no significant intergroup differences was observed at 3 and 5 years, which may be attributable to dynamic renal function and recipient body weight change. We observed no significant intergroup differences in the complications rates. This observation indicates no difference between adult and pediatric recipients using kidneys obtained from extremely low body weight donors.

Based on lessons learned from previous failures, we did not perform dual separating kidney transplantation in cases of kidneys obtained from donors with body weight ≤5 kg and also adopted an improved surgical technique of en-bloc kidney transplantation during which the distal abdominal aorta was used as the outflow tract and was anastomosed to the external iliac or inferior epigastric artery of the recipient, thereby minimizing the risk of thrombosis and postoperative bleeding without the administration of conventional anticoagulants postoperatively (16, 37). We also modified the operative procedure and protected the arterial sheath of the transplanted kidney based on the following rationale: (a) The graft artery wall receives a part of its blood supply from the vascular sheath. Complete separation of the renal artery sheath may cause ischemic contracture of the renal artery wall. (b) It is important to maintain the integrity of the graft renal artery sheath to ensure adequate blood supply for the growth of the renal artery and to improve the quantity of antibiotics delivered to the renal vascular wall to prevent infection-induced vascular rupture. (c) The graft artery sheath supports small renal vessels and prevents distortion and angling of the graft artery.

We categorized the donor weight ≤5 kg group into the novel operation and conventional operation subgroups (including en-bloc kidney and dual separating kidney transplantation). The thrombosis, DGF, hydronephrosis, and ureteral stenosis rates were lower in the novel operation than in the conventional operation group; however, the difference was statistically nonsignificant. The urine leak rate was significantly lower in the novel operation than in the conventional operation group, without any significant intergroup differences in patient survival, graft survival, and death-censored graft survival rates which might attribute to the fact that during back- table graft preparation tissue surrounding ureter has been well conserved for better ureter blood supply. This suggests that the novel operation offers certain advantages over the conventional operative approach.

In our opinion, the results of the novel transplantation method described in our study are replicable and may be further improved over time; however, large-scale studies with long-term follow-up are warranted to validate our results. In this study, the lowest weight of donors was 1.9 kg and the kidneys of which are transplanted to a 12-years-old male child with the novel surgery method. Thus far this recipient has been followed up for 5 years with the kidney allograft functioning. We intend to perform further research with large-scale studies that include longer follow-up to investigate graft allocation policies, graft growth, blood pressure control, immune suppression, and other relevant issues in this domain.

## Conclusion

To summarize, graft survival rates of kidney transplantation using kidneys from donors of extremely low body weight ≤5kg were lower than those from donors of greater body weight. However, renal function was unaffected by differences in donor body weight. Donor body weight ≤5kg group is at elevated risk of graft loss caused by thrombosis and acute rejection. In our view, the outcome of extremely low body weight donor kidney transplantation is independent of whether the recipient is adult or child. And the novel operation mentioned in this study may also reduce complication rates. Although there are still many challenges in kidney transplantation of donor body weight ≤5kg, it can be improved through technical improvement such as the novel operation proposed by our center. In conclusion, donors with body weight ≤5 kg can be considered as an useful source to expand the donor pool to enable treatment of a larger number of patients with end-stage renal disease.

## Data Availability Statement

The datasets presented in this article are not readily available because of participants identifiable data. Requests to access the datasets should be directed to Longkai Peng, penglongkai@csu.edu.cn.

## Ethics Statement

The studies involving human participants were reviewed and approved by Ethics Committee of the Second Xiangya Hospital of Central South University. The patients/participants provided their written informed consent to participate in this study.

## Author Contributions

JP drafted the manuscript. JP, MZ, ZT, TL, SS, HY, and JH collected data. RH designed statistical method. JP and MZ generated the figures. LP, XX, SY, FP, GL, YW, and XT performed the surgery. SY revised the manuscript. LP, HD, and HZ designed the outline of the manuscript and revised the manuscript. All authors contributed to the article and approved the submitted version.

## Funding

This work is supported by the National Science Foundation of China (82070776, 81900370, 81800664 and 81970655), Excellent Youth Foundation of Hunan Province of China (2021JJ10076) and Huxiang Young Talents of Hunan Province (2019RS2013).

## Conflict of Interest

The authors declare that the research was conducted in the absence of any commercial or financial relationships that could be construed as a potential conflict of interest.

## Publisher’s Note

All claims expressed in this article are solely those of the authors and do not necessarily represent those of their affiliated organizations, or those of the publisher, the editors and the reviewers. Any product that may be evaluated in this article, or claim that may be made by its manufacturer, is not guaranteed or endorsed by the publisher.
